# Effect of Nanosecond Laser Texturization on Tribological Behavior of AISI 321 Stainless Steel

**DOI:** 10.3390/ma17235870

**Published:** 2024-11-29

**Authors:** Paweł Zawadzki, Sergey Dobrotvorskiy, Borys Aleksenko, Rafał Talar

**Affiliations:** 1 Faculty of Mechanical Engineering, Poznań University of Technology, Piotrowo 3, 61-138 Poznań, Poland; 2 National Technical University “Kharkiv Polytechnic Institute”, 2, Kyrpychova Str., 61002 Kharkiv, Ukraine; 3 Poznań University of Life Sciences, Wojska Polskiego 38/42, 60-637 Poznań, Poland

**Keywords:** tribology, nanosecond pulsed laser, stainless steel, AISI 321, surface roughness, surface modification, LIPSSs, surface hardness

## Abstract

This study investigates how laser-induced surface modifications influence key properties such as wear resistance, hardness, and friction in dry and lubricated conditions. The research applies nanosecond pulsed laser treatment to create random, quasi-random, quasi-periodic, and periodic surface structures on the steel surface, aiming to enhance the wear resistance and reduce the coefficient of friction (COF). The frictional performance between the carbon steel ball and the texturized surface was evaluated, including an analysis of the initial friction phase contact (single, double, and multi-contact), with the surface topography assessed before and after wear. The results of the pin-on-plate tests indicate that laser texturing improves the hardness by transforming austenite into martensite, modifies the wettability by periodizing the surface, reduces the COF, and enhances the wear resistance. Periodic surface structures allow for better lubricant retention and change in the lubrication regime, contributing to lower friction and a longer surface lifespan. Minimizing ball–surface contact through appropriate surface periodization significantly affects the load transfer. The primary wear phenomena are the adhesive and abrasion wear of a two-body nature, transforming into a three-body one. The study concludes that laser surface texturing is an effective method for enhancing the tribological performance of AISI 321 steel, with potential applications in industries requiring high wear resistance.

## 1. Introduction

Recent advancements in material science, surface engineering, and computational techniques are revolutionizing tribology by enhancing industry performance and sustainability. Innovations such as advanced coatings, novel materials, and machine learning address the challenges of friction, wear, and lubrication. AISI 321 stainless steel, known for its corrosion resistance and mechanical properties, faces difficulties in friction and wear due to a low surface hardness and susceptibility to abrasion under tribological conditions. Untreated AISI 321 exhibits significant wear, limiting its use in high-friction environments [1]. Its tribological properties—wear resistance, hardness, and frictional behavior—are critical in industries like chemical processing, food production, and construction [2,3]. Surface treatments, including laser processing and coatings, improve these properties by modifying the microstructure and phase composition [4].

Laser texturization modifies the surface texture, phase composition, and hydrophobicity, making materials suitable for fluid management and mechanical applications. Nanosecond lasers, emitting pulses in the nanosecond range, interact with materials through rapid heating, melting, and vaporization, creating intricate two-scale periodic structures [5,6]. This method involves using a nanosecond pulsed laser, effectively modifying the surface morphology of materials such as steel, enhancing their hydrophobic properties [7]. In tribological applications, nanosecond laser texturing significantly reduces the coefficient of friction (COF) in lubricated conditions by promoting hydrodynamic lubrication through micro-hydrodynamic effects [8]. Additionally, it forms stiff bulges around patterns, which decrease friction under lubricated conditions despite increasing it in dry conditions [9]. The ability to design specific patterns, such as crosshatches or dimples, makes this technique versatile for optimizing tribological properties.

The laser structuring of stainless steel, particularly AISI 321, involves advanced techniques to enhance its surface properties, primarily for improved tribological performance. The process typically employs laser systems to create laser-induced periodic surface structures (LIPSSs) [7], which are known for their uniformity and efficiency in reducing the COF and wear on stainless steel surfaces [10]. The laser structuring process can significantly refine the microstructure of the stainless steel surface, as seen in the case of AISI 321, where Surface Mechanical Attrition Treatment (SMAT) was used to promote the nucleation of sulfides and refine the grain size from 600 µm to 100 µm, resulting in a nanocrystallized layer with equiaxial nanograins of about 21 nm [1]. This refinement enhances the surface’s mechanical properties and improves the wear resistance. The application of laser structuring on AISI 321 also involves symmetry modifications in the surface’s morphology and chemistry, which are analyzed using Scanning Electron Microscopy (SEM) and energy-dispersive X-ray Spectroscopy (EDS) [11]. These modifications are crucial for achieving lower COF values, as demonstrated in dry sliding tests using a block-on-ring arrangement, where the LIPSS-treated surfaces showed significantly reduced friction compared to the untreated surfaces [11]. Furthermore, Nonlinear Laser Lithography (NLL), an advancement over traditional LIPSSs, offers improved uniformity and long-range order, allowing for high-speed processing over large areas. This method has shown that, in lubricated conditions, the COF values of NLL-treated surfaces are nearly half of those of untreated surfaces, with even more significant reductions observed in dry conditions [12]. The laser structuring process is versatile, allowing for the creation of various texture morphologies, such as dimples, grooves, and sinusoidal patterns, which can be tailored to specific applications to optimize the tribological behavior [13]. These textures are generated using precise laser parameters, such as the wavelength, power, and scanning speed, to achieve the desired surface characteristics. Overall, the laser structuring of AISI 321 stainless steel is a sophisticated process that significantly enhances its surface properties, making it suitable for applications requiring high wear resistance and low friction, such as in chemical, biomedical, and nuclear industries [12,13].

Laser structuring can significantly reduce the friction coefficient of surfaces. For instance, studies on diamond-like carbon (DLC) coatings have shown that laser interference patterning can reduce the friction coefficient from 0.18 to 0.11, achieving values comparable to lubricated surfaces [14]. This reduction is crucial for applications where lubrication is undesirable or impractical. The orientation of laser-induced periodic surface structures (LIPSSs) can lead to anisotropic frictional properties. Structures oriented perpendicular to the sliding direction have been shown to reduce the energy consumption and improve the wear resistance compared to parallel or non-textured surfaces [15]. This anisotropy can be strategically used to optimize the frictional performance in specific applications. Laser structuring enhances the wear resistance by creating surface patterns that evenly distribute contact stresses. For example, LIPSSs on stainless steel have been shown to significantly reduce wear in tribological tests, demonstrating the potential for extending the lifespan of components [16]. The laser structuring process can also induce surface hardening, improving the wear resistance. This is particularly beneficial in high-stress applications where surface degradation is a concern [10].

Creating periodic micro- and nanoscale structures on steel surfaces, such as laser-induced periodic surface structures (LIPSSs), enhances the tribological properties like friction and wear resistance. LIPSSs on AISI 321 stainless steel improve the performance by modifying the surface characteristics, with the orientation and periodicity of structures influencing the anisotropic behavior [15]. Periodic microstructures affect the contact mechanics in lubricated and dry conditions, with geometry (e.g., ball-on-disk vs. linear motion) impacting the stress distribution and wear patterns. For example, ball-on-disk tests showed increased wear and oxidative effects, acting as microlubricants [17]. Surface roughness also plays a crucial role; rougher surfaces (*Sa* = 0.61 μm) showed lower wear under lubrication compared to smoother ones (*Sa* = 0.08 μm) [18]. In fretting conditions, rough surfaces require less energy to activate wear modes than smooth surfaces. The transition from partial to entire slip regimes is influenced by the surface morphology, affecting the wear dynamics and damage modes such as fretting cracking and wear [19]. The surface texture, such as square and round pit-textured surfaces, can alter the tribological behavior. Round pit-textured surfaces (RPTSs) are surfaced with regularly arranged circular depressions (pits) that enhance the tribological properties and better suppress friction-induced vibrations than other textures despite the slightly higher contact resistance. This suggests that specific textures can optimize the tribological performance by modifying surface interactions [20]. The roughness of substrates also impacts the performance of coatings. For example, DLC coatings on rough substrates showed increased wear rates and potential for delamination due to high asperity levels, hindering the protective transfer film formation [21]. At the nanoscale, roughness influences tribofilm formation, which affects the friction and wear dynamics. Initially, rough surfaces control sliding resistance, but wear particle agglomeration shifts the friction to depend on the actual contact area, aligning with contact plasticity theory [22].

AISI 321 stainless steel faces significant challenges in high-friction and wear environments due to its relatively low surface hardness and susceptibility to severe abrasion, particularly under tribological conditions. While laser surface texturing methods exist, the wear mechanisms and their interaction with surface structures require further investigation. This study subjected AISI 321 steel to nanosecond laser texturing, thoroughly examining its hardness, wettability, and surface topography. The hypothesis posits that laser structuring can enhance the tribological properties by improving the surface periodicity. However, the frictional performance depends significantly on the contact conditions, such as the presence or absence of lubrication. Tribological tests were conducted to assess the treated surface, analyzing the initial contact mechanics, the COF, and the wear behavior. Despite advancements in surface engineering, there are the following critical research gaps: an insufficient understanding of the nanoscale interactions between the surface roughness, wear particles, and contact area; limited data on the long-term behavior of laser-textured surfaces under dynamic or mixed lubrication regimes; incomplete knowledge of the anisotropic properties of LIPSS structures and their effect on the wear resistance and energy efficiency. The influence of laser parameters, such as the wavelength, power, and scanning speed, on the performance remains inadequately explored. A lack of systematic studies on optimal structures (e.g., pits, grooves, or LIPSSs) for various lubrication and dry conditions further highlights the need for focused research. This study aims to address these gaps, providing innovative insights into enhancing the tribological properties of AISI 321 stainless steel through laser texturing.

## 2. Materials and Methods

### 2.1. Laser Surface Texturing of AISI 321

**Figure 1:**
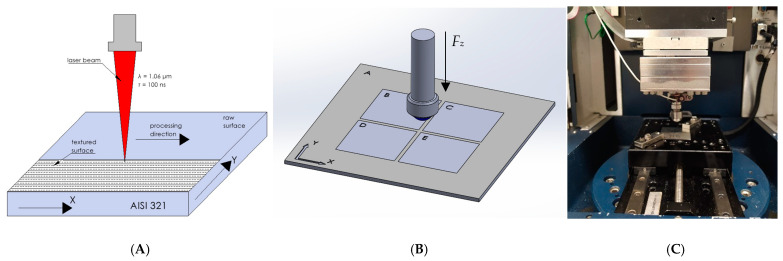
The scheme of (**A**) the laser texturing, (**B**) the sample and ball motion used during tribological tests (*F_z_*—pressure force), and the test stand—a Bruker UMT (**C**).

AISI 321 stainless steel consisting of 17% to 19% chromium, 9% to 12% nickel, and 0.08% carbon was used for testing. It also contains up to 2% manganese, silicon, and titanium. The steel used was a piece of steel sheet produced by rolling. The surface was not subjected to any additional mechanical processing. Five alloy surfaces were prepared, marked as follows: A (raw surface), B, C, D, and E (laser-texturized surfaces) (Figure 1A,B). Based on the ytterbium fiber laser, the texturing process used a Minimarker-2 unit (Saint Petersburg, Russia). The following parameters were used during the process: wavelength, *λ* = 1.06 μm; pulse duration, *τ* = 100 ns; average pulse power, *P_apg_* = 1–50 W; pulse repetition rate, *f* = 10–99 kHz; laser beam diameter, *d*_0_ = 25 µm; a Gaussian beam profile. The AISI 321 steel plate was irradiated at four sections with laser pulse powers equal to *P_15%_ =* 7.5 W (Figure 1B (B)), *P_20%_ =* 10 W (Figure 1B (C)), *P_30%_ =* 15 W (Figure 1B (D)), and *P_40%_ =* 20 W (Figure 1B (E)).

### 2.2. Surface Characteristics Measurement

The surface of the samples was assessed in terms of the morphology using the Alicona IF-Portable RL optical profilometer (Billerica, MA, USA), and the profile and roughness parameters were analyzed. Profilometry analyses covered a 1.014 × 1.014 mm square area in the central part of the sample. Atomic Force Microscopy (Bruker, Billerica, MA, USA) measurements were conducted with an ICON Scanning Probe Microscope in air, at room temperature, using tapping mode with TESPA probes at a scanning rate of 0.2 Hz and a resolution of 512 × 512 pixels. AFM data analysis was performed using the NanoScope Analysis 1.5 software. Hardness tests were conducted on the microhardness tester FM-700 (Tokyo, Japan) for both the base alloy and the laser-textured surface using the Vickers method with a load of 5 kg and a dwell time of 7 s, with five measurements taken per sample. Additionally, spectroscopic measurements of the steel surface composition on the XRF Fisher Spectrometer (Sindelfingen, BW, Germany) were carried out before and after the steel texturization.

### 2.3. Wettability Test

The research used the Ossila Contact Angle Goniometer (Sheffield, UK) using demineralized water. The device features a measurement accuracy of ±1° and a range of 5° to 180°. With compact dimensions of 95 mm × 165 mm × 320 mm and a working area of 50 mm × 50 mm, the goniometer is easy to operate and ideal for measuring samples with a maximum thickness of 20 mm. Additionally, being equipped with a camera offering a maximum resolution of 1920 × 1080, it allows for the accurate recording of results.

### 2.4. Tribology Test

Following the modified ASTM standard [23], the tests were performed in the pin-on-plate variant using the UMT-2 Tribolab (Billerica, MA, USA) laboratory tribotester (Figure 1C). The test was carried out under a constant force of 1 N and 3 N at a speed of 10 mm/s for 360 s (Table 1). The measuring section was 5 mm. The test object was screwed to the machine table to prevent its movement during the test. A 5 mm diameter bearing ball, made of 100Cr6 steel, was mounted longitudinally to a *Z*-axis. The sample’s surface and the balls were cleaned using methanol to remove contamination. The tests were performed in a laboratory with a constant temperature of 21 °C and 50% humidity. The tests were carried out in dry conditions and conditions of lubrication with silicone oil: XIAMETER^TM^ PMX-200 with a viscosity of 10cSt. The oil was applied directly to the steel surface during the test.

During the test, the bearing ball was in constant contact with the sample surface, making a reciprocating motion along the Y- and X-axis of the machine. After each test (one track), the ball was replaced with a new one. The machine’s software measured and recorded the current position, friction coefficient, and the force applied to the sample surface throughout the test.

## 3. Results and Discussion

### 3.1. Surface Topography Evaluation

According to research conducted by Liang et al. [24], the surface roughness can alter the distribution of contact pressures, which, in turn, affects the frictional response of the material. This occurs because rough surfaces generally have a smaller actual contact area than smooth surfaces, leading to higher contact pressures at the asperity peaks.

**Figure 2:**
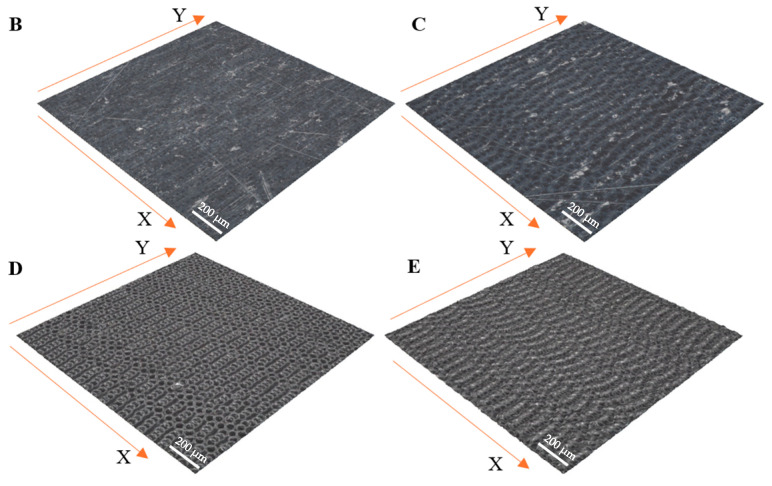
Isometric view of the surface topography after laser surface texturing before the tribology test with laser scanning directions (X and Y).

Figure 2 and Table 2 present the surface topography measurements of the texturized samples. The interaction of a nanosecond laser beam with the AISI 321 surface involves heating, melting, evaporation, and particle formation. Increasing the laser power intensifies the indicated processes, accelerating the structuring. The intensification of evaporation results in an increase in the depth of the valleys. On the other hand, the intensification of melting causes the formation of outflows, causing an increase in the height of the roughness peaks. The combination of both phenomena results in the development of the surface topography.

Sample A exhibits the lowest values of Sa (0.24 µm) and Sq (0.31 µm), indicating a relatively smooth surface. However, the high Sp (6.04 µm) and Sv (42.4 µm) values suggest significant extreme features, such as peaks and valleys, which could lead to increased wear due to the entrapment of abrasive particles. Additionally, the high Sku value (412) implies that the surface has numerous extreme peaks and valleys, potentially affecting the friction stability. Sample B shows slightly higher Sa (0.25 µm) and Sq (0.34 µm) values, indicating greater surface roughness than the raw sample. However, lower Sp (3.23 µm) and Sv (33.8 µm) values suggest fewer extreme features, which may result in more predictable friction and reduced wear. The negative Ssk value (−5.25) indicates the dominance of valleys on the surface, which could improve the lubricant retention.

Sample C demonstrates higher Sa (0.28 µm) and Sq (0.34 µm) values, suggesting increased surface roughness. However, very low Sp (1.32 µm) and Sv (1.6 µm) values indicate a more uniform surface with fewer extremes, which may lead to reduced wear and more stable friction. This sample exhibits the most predictable behavior under tribological conditions, as evidenced by the low S10z (2.48 µm) and Sku (3) values. Sample D is characterized by higher Sa (0.38 µm) and Sq (0.49 µm) values, suggesting greater surface roughness. Moderate Sp (2.96 µm) and Sv (7.7 µm) values indicate a more balanced surface with moderate height variations. The lower Sz (10.6 µm) and S10z (5.60 µm) values suggest a more uniform surface, which could result in more stable friction and reduced wear.

Sample E shows the highest Sa (1.00 µm) and Sq (1.23 µm) values, indicating the roughest surface. The Sp value (9.38 µm) is the highest, suggesting the presence of pronounced peaks, while the low Sv value (3.1 µm) indicates fewer deep valleys. This sample exhibits the most significant roughness, possibly leading to higher initial friction. However, due to the higher Sdr value (2.42%), the surface has a more complex topography that may better retain lubricant, potentially reducing the wear over time. The high Ssk value (0.44) suggests a dominance of peaks, which influences the lubricant retention and friction characteristics.

**Figure 3:**
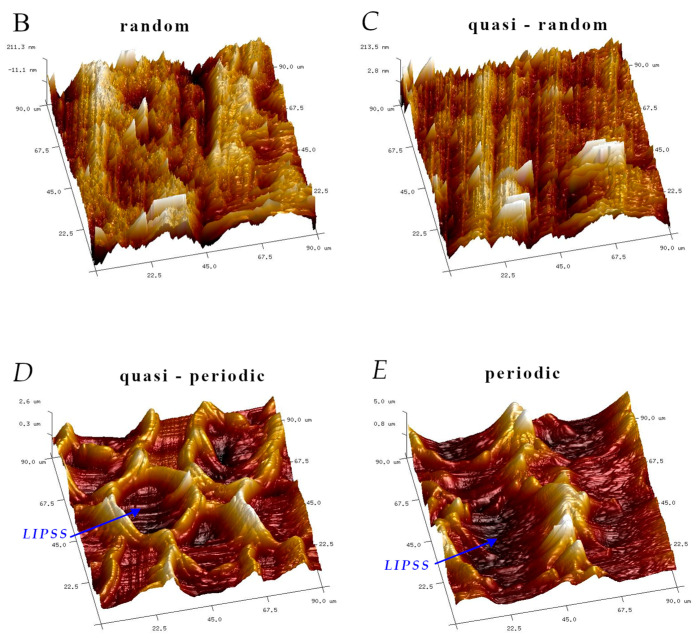
Results of the surface topography measurements using the AFM method. LIPSSs—laser-induced periodic surface structures: B—random roughness [25], C—quasi-random roughness, D—quasi-periodic roughness [26], and E—periodic roughness [27].

The analysis of the microscope images obtained using the AFM method reveals the impact of structuring on the surface topography of steel on the micro- and nanoscales (Figure 3). The micrometer scale is visible on surfaces D and E, where periodic surface structures are noticeable. For sample E, the periodic structures are aligned parallel to the Y-axis and perpendicular to the X-axis. Due to the distinct nature of the topography, the periodic structures for sample D do not maintain a regular distribution. From a tribological perspective, this is significant due to the contact potential, especially in the initial friction stages. Micrometric changes are also noticeable on surfaces B and C, where radiation interaction has led to the flattening and homogenization of the surface. LIPSSs are evident along the laser beam scanning direction in periodic grooves. Similar to samples D and E, these features are significant during surface contact but can also provide an excellent basis for forming an oil film.

Based on the analysis of the surface topography, the following textured surfaces can be identified: B—random [25], C—quasi-random, D—quasi-periodic [26], and E—periodic [27] (Figure 3).

For instance, studies on micro-textured aluminum–silicon alloys have shown that more negative Ssk, higher Sku, and more excellent Sv (depth of the deepest indentation) values can lead to lower friction coefficients with constant Sa and Sq values. Using textures similar to those in samples 3 and 4 could reduce the wear due to the better lubricant retention and stress dispersion [28]. Additionally, studies on nickel–aluminum alloys indicate that different roughness parameters affect the wear at high temperatures, with regular surface patterns reducing the friction and wear through the formation of tribological layers [29]. For example, in the study by Chen et al. [28], more negative Ssk values and higher Sku values can lead to lower friction coefficients. In other studies, such as those by Zhang et al. [30] and Zhong et al. [31], by optimizing these parameters, the surface texturing can significantly impact the friction and wear. Matikainen et al. [32] emphasize that different mechanical processing techniques, such as laser texturing, can improve the tribological properties by modifying the surface roughness.

### 3.2. Hardness

**Figure 4:**
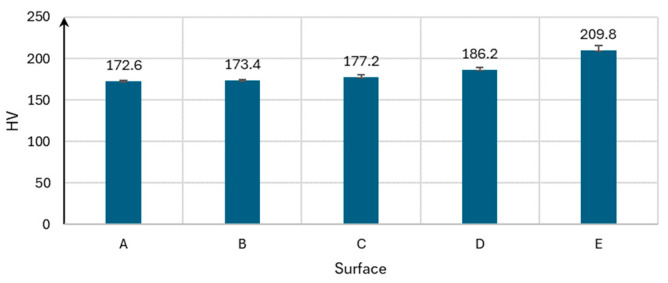
The hardness of the surface samples: A (raw surface), B, C, D, and E (laser-texturized surfaces).

The Vickers hardness test was conducted on five different surfaces subjected to laser structuring at various laser powers. The results show that the surface hardness increases with an increasing laser power (Figure 4). The possible reasons for this include chemical reactions leading to the formation of oxides, phase transformations, and a grain size reduction in the material’s structure. Spectroscopic analysis showed the following percentage changes in the Fe content relative to the base surface, respectively: (B) −1.5%, (C) −1.4%, (D) −2.1%, and (E) −0.5%; for Cr: (B) −1.6%, (C) −2.4%, (D) −1.3%, and (E) −1.4%. Changes in the contents of the remaining components, Ni, Mn, Mo, and Ti, are within the measurement error range and do not show any dependence on the processing level. The thermal cycle induced by the laser treatments significantly impacts the evolution of the microstructure. Rapid heating and cooling rates can lead to the formation of complex microstructures, including the coexistence of austenite and martensite phases [33,34]. Repeated laser scanning can stabilize austenite in carbon-bearing steels through carbon partitioning, which affects the martensitic transformation upon cooling [35]. High cooling rates after laser treatment contribute to martensite formation, a more complex phase than austenite. This transformation is crucial for improving the mechanical properties of the material. In laser surface treatments, martensite formation is often observed in the heat-affected zones, contributing to enhanced wear resistance [36,37]. As the hardness increases, the level of material wear should decrease [38], although the COF and the level of counter-specimen wear may also increase [39].

### 3.3. Wettability

**Figure 5:**
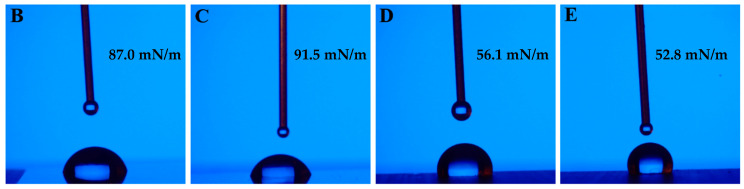
Wettability test results for the laser-texturized surfaces with surface energy values.

Surface energy affects the material’s ability to absorb or spread liquids. It also influences the interactions between microscopic asperities (protrusions) on the surfaces. Surfaces with a high surface energy may have stronger interactions between asperities, which can lead to higher friction. Based on wettability characteristics, the samples can be divided into groups (B and C, and D and E; see Figure 5). This will be used in further analysis, including the wear. However, it is significant that lower wear was observed for surfaces with a lower wettability, confirming the following relationship: low wettability–high friction resistance. Superhydrophobic surfaces, characterized by high water contact angles, exhibit lower friction coefficients due to the reduced adhesion between the surface and the contacting material. Conversely, superhydrophilic surfaces with low water contact angles can enhance the wear resistance by promoting better lubrication. This is because the increased wettability facilitates the formation of a stable lubricant film, reducing the direct contact between the surfaces and the wear [40]. Textured surfaces with sinusoidal patterns have been shown to reduce friction by creating a hydrophobic layer that minimizes direct contact and wear [40].

### 3.4. Surface Topography After Test Evaluation

The topography of the sample surfaces changed after performing the tribological tests. The changes can be divided into the following two groups: (I) surface development through the exchange of material between the sample surface and the ball, and (II) sample surface flattening through material removal. In the case of the raw surface, two-body abrasive wear is observed, where a scratch is formed due to plastic deformation. In subsequent stages, the resulting debris causes further material wear, intensifying the process and transitioning the system to three-body abrasive wear [41]. Three-body abrasive wear is caused by particles entrapped between two sliding surfaces.

**Figure 6:**
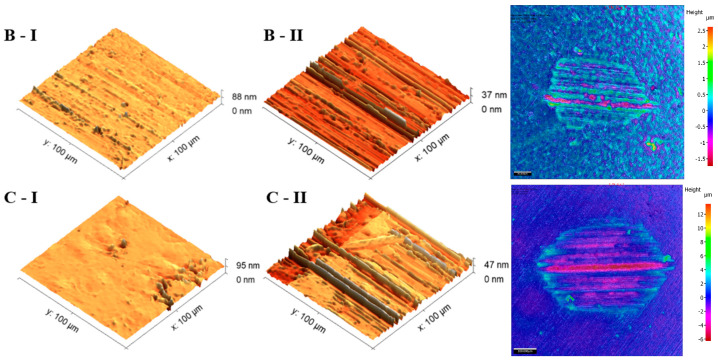
AFM visualization of the surfaces (B and C) before (I) and after (II) the tribology test under a 1 N load and with lubricant, and the ball wear level.

The first phenomenon (I) involves surfaces B and C. Material is transferred from the ball to the sample surface, and this material adheres to the flat surface of the sample due to adhesion [42]. In the observed case (Figure 6), the transfer occurs from the ball surface to the sample surface, as evidenced by the distinct wear on the ball’s structure and the excess material on the sample’s surface. No plastically deformed scratch is formed. As a result of laser texturing, the hardness of surfaces B and C increased, which resulted in the higher wear of the ball surface. This is confirmed by the hardness tests (Figure 2) and the literature reports by Rahman et al. [43] or Jazdzewska et al. [44]. Similar observations were noted for both *F_z_* = 1 N and 3 N. No influence of laser structuring on the AFM analysis results was observed for samples B and C. The surface topography of samples B and C undergoes visible changes with an ambiguous trend. However, noticeable differences are observed between the X and Y directions for sample B in the parameters Sz, Ssk, and Sku (see Table 3). The Sku parameter drops below three, indicating a height distribution above the average plane. The decrease in Sz suggests surface flattening. The reduction in Ssk values indicates increased symmetry around the average plane. For sample C, the number of parameter values it remains ambiguous.

**Figure 7:**
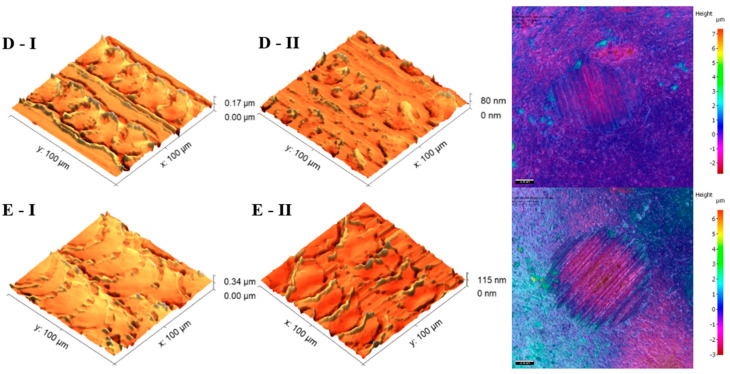
AFM visualization of surfaces (D and E) before (I) and after (II) the tribology test under a 1 N load and with lubricant, and the ball wear level.

Typical two-body abrasive wear phenomena were observed for surfaces D and E, where the surface structure exhibits a periodic arrangement. The surface topography undergoes distortions (Figure 7). All of the topography parameter values for surface D decreased, regardless of the conditions and orientation direction. Apart from the flattening of the structure indicated by decreases in Sa, Sq, and Sz, the decrease in Ssk suggests a reduction in severities, and the decrease in Sku indicates a lower sharpness. The decline in Vvc values is likely due to debris filling the core void volume. Similar observations were noted for surface 4.

The presence of oil reduces the degree of changes in the surface topography, similar to a lower Fz. However, oil lubrication has a higher influence on the changes. The difference in the extent of the changes indicates less distortion of surfaces D and E.

### 3.5. Coefficient of Friction

Comparing the average COF values for the contact between the ball and the laser-textured surfaces in the steady state with the baseline surface A, it can be observed that, for all of the surfaces, there is a decrease in the COF regardless of the direction (Table 4). Exceptions are found for surfaces C, D, and E when *F_z_* = 1 N in the Y direction in the presence of oil. In the X direction, oil lubrication reduces the COF for all of the analyzed surfaces, regardless of *F_z_*. A general analysis of the COF for the textured surfaces indicates that significantly higher COF values were recorded at *F_z_* = 1 N compared to *F_z_* = 3 N. However, the most significant percentage decreases in the COF compared to surface A were observed at *F_z_* = 1 N under dry conditions and *F_z_* = 3 N under oil lubrication. An increase in *F_z_* from 1 N to 3 N generally reduces the COF values, except for surfaces D and E under oil lubrication in the X direction, where no changes were noted.

It was also observed that lower COF values were recorded under dry conditions with *F_z_* = 1 N compared to oil lubrication. Interestingly, the opposite trend was noted for *F_z_* = 3 N. This phenomenon may result from a reduction in the sliding friction (at *F_z_* = 1 N) due to the formation of a layer between the contact surfaces in favor of increased rolling friction due to hydrodynamic effects, as also observed by Popescu et al. [45]. Since these changes were not observed for surface A, it can be assumed that texturing significantly influenced the frictional properties of the steel–steel contact.

Surface properties can modulate the effectiveness of hydrodynamic lubrication, potentially leading to increased friction under certain conditions, as also indicated by studies conducted by Hirayama et al. [46]. Additionally, the distinct difference in the COF characteristics for the two load values highlighted the threshold of these effects.

**Figure 8:**
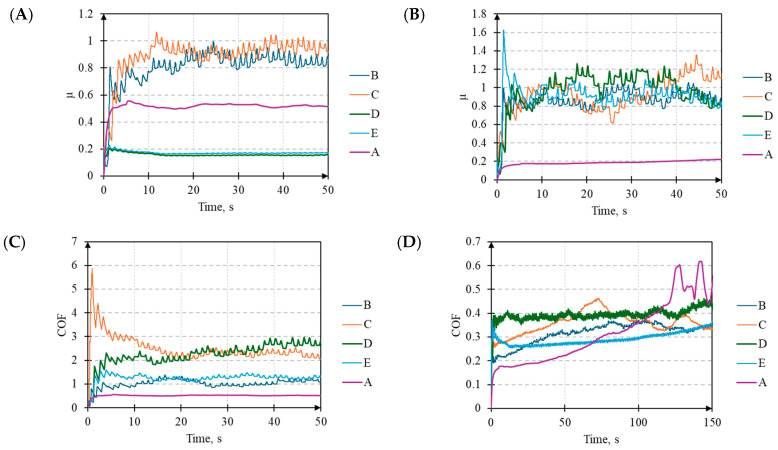
The time evolution of the COF values for *F_z_* = 1 N under the following conditions: (**A**) direction X and oil lubrication, (**B**) direction X and dry conditions, (**C**) direction Y and oil lubrication, and (**D**) direction Y and dry conditions for surface samples: A (raw surface), B, C, D, and E (laser-texturized surfaces).

Furthermore, differences were observed in the time evolution of the COF values, for which the following two distinct stages can be identified (Figure 8): the initial stage and the stabilized stage. In all cases with *F_z_* = 1 N for surface A, the COF initially increases and then stabilizes at a value higher than the initial one. A distinct behavior was observed for surface C under oil lubrication in the Y direction and surface E under dry conditions in both directions. Surfaces D and E under oil lubrication in the X direction are characterized by low change fluctuation. The other cases for surfaces B, C, D, and E show significant COF fluctuations, which are notably higher in the X direction under dry conditions. An atypical stabilized stage was observed for surfaces B and C under dry conditions in the Y direction. The moment of transition from a low COF to a high COF, and its subsequent stabilization, is prolonged.

**Figure 9:**
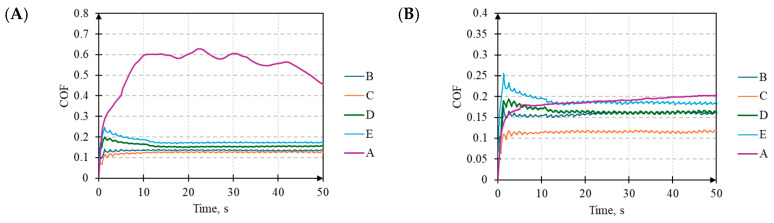

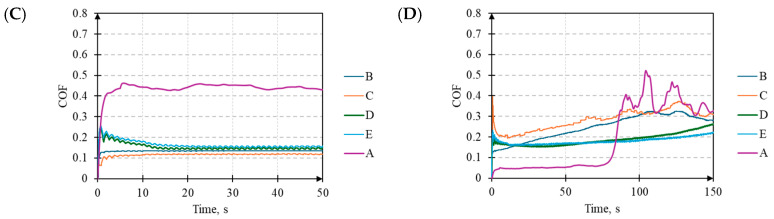
The time evolution of the COF values for *F_z_* = 3 N under the following conditions: (**A**) direction X and oil lubrication, (**B**) direction X and dry conditions, (**C**) direction Y and oil lubrication, and (**D**) direction Y and dry conditions for surface samples: A (raw surface), B, C, D, and E (laser-texturized surfaces).

For *F_z_* = 3 N, the COF evolution for surface A also features a sudden increase in the initial stage, similar to *F_z_* = 1 N (Figure 9). However, under dry conditions, the duration of the initial stage is significantly longer. It includes an apparent stabilization that, due to the sudden destruction of the ball and surface, transitions into a chaotic yet steady stabilized stage. The COF values under oil conditions for surfaces B, C, D, and E are similar regardless of the direction (X or Y). The difference appears in the initial stage, as surfaces D and E exhibit an increase in the COF, followed by a decrease to a stabilized value. A similar pattern is observed under dry conditions. In this case, the initial phase is characterized by a higher COF, as the asperity peaks undergo plastic deformation and micro-welding, influenced by the periodicity and amplitude of the surface roughness [47].

Referring to the average COF values and the presented graphs, it can be concluded that, under low-load conditions (*F_z_* = 1 N), laser texturing in clearly defined conditions can reduce the COF and alter its evolution. For higher *F_z_* values, texturing seems to always have a positive impact on the results and stabilizes the changes. Similar to the studies mentioned above, the analyses conducted by Wang et al. [21] and Bahshwan et al. [48] on textured surfaces further confirms the view that surface modifications can significantly influence the tribological performance. They found that round surfaces with dimpled textures exhibited the lowest coefficients of friction and friction forces, demonstrating the potential of surface texturing to optimize the contact conditions and reduce the friction-induced vibrations.

From the perspective of the complexity of the stabilization process, the COF evolution under dry conditions in the Y direction for both *F_z_* = 1 N and *F_z_* = 3 N is particularly interesting. For surfaces D and E, the COF behavior exhibits characteristics similar to those observed previously. However, for surfaces B and C, a noticeable continuous increase in the COF is observed until stabilization is reached. In this specific case, the direction of the ball movement relative to the surface structure, as well as the structure of the surface itself, likely influences the COF evolution.

Based on the analyses performed, it can be concluded that the surface structure plays a more significant role in the context of the COF. The surface structure directly affects the contact mechanics and the ability to store lubricant, which has a key impact on the friction behavior. Although lubrication also significantly modifies the interactions between surfaces, the surface structure determines the initial conditions that determine the effectiveness of the lubricants.

### 3.6. Wear on a Cross-Section of Steel

Analysis of the scratch width in the X and Y directions does not indicate a significant directional effect of the structure on the wear, which suggests a lack of clear anisotropy in the tested samples. Table 5 describes the wear mechanisms in the stabilized state (II), where, in the case of non-periodic surfaces (B and C), a decrease in the wear is observed with a decreasing load and the addition of lubricant. Much less obvious decreases are observed for the periodic surfaces D and E. However, the width of the wear path is not the only determinant of the tribological properties because, as shown in the following tables, the impact on the wear is exerted by contact at the level of the individual peaks. The base surface (A) shows the most significant susceptibility to wear. In contrast, the periodic surfaces (D and E) differ in their resistance, which may result from differences in the structure and load distribution capabilities.

For surface B (Table 6), regardless of the structural orientation, an expansion of the cross-sectional structure is observed at the path’s location, which confirms an increase in the *Sa* and *Sq* parameters (Table 4). Cracks (Table 6 (A)) and material deposits (Table 6 (B)), remnants of the destruction of the surface roughness peaks, appear. Under dry conditions, with *F_z_* = 3 N, the phenomenon of the initial two-body abrasive wear is observed, which transitions into adhesive wear due to the formation of a wear film [48,49]. In other cases, two-body abrasion wear [50] is observed, which, due to the noticeable presence of free material fragments (see Table 6 (B)), can develop into three-body abrasion [50]. The oil film formed in this process thus reduces the level of wear.

For surface C, more intense abrasive wear was observed under dry conditions. However, no influence of directionality was noted (see Table 7). Cross-sectional analysis indicates surface expansion in the form of material deposits with numerous fine scratches. The addition of oil reduces the level of wear, although, for *F_z_* = 3 N, a greater amount of material deposit was observed compared to surface 1. The absence of deformation on the sample surface suggests that the material deposit originated from the ball. The wear mechanisms are the same as those observed for surface B.

For sample D, due to the complexity of the structure, a more significant number of cross-sections were distinguished (see Table 8). Owing to the periodicity of the structure, changes in the cross-sectional profile are noticeable, such as the distortions of the peaks. The dominant phenomenon is abrasive wear. The most significant surface distortions were observed under dry conditions with *F_z_* = 3 N. However, the structure described as a “honeycomb” (purple color) exhibited more pronounced deformations than the regular structure. The cross-sections indicate the reduced susceptibility to wear in the case of *F_z_* = 1 N and oil lubrication.

Surface E exhibits the lowest surface structure wear (see Table 9) compared to the other samples, regardless of the structural orientation (X or Y). Damage is primarily noticeable under dry conditions at *F_z_* = 3 N, where it takes the form of scratches along the direction of the ball movement. In other cases, no significant changes were observed.

Based on the analysis, it can be concluded that the periodicity of the structure significantly influences the cross-sectional changes and surface wear. Periodic structures, such as those observed on surface D, may distribute the load more effectively and reduce wear under oil lubrication conditions, which is supported by the literature highlighting the beneficial impact of periodic structures on the tribological properties [51]. Irregular or less periodic structures may lead to increased wear. These findings underscore the importance of proper surface structure design to minimize the wear and enhance the material durability in tribological conditions.

### 3.7. Frictional Performance of the Carbon Steel Ball

The normal force *F_z_* has a significant effect on the wear of the ball. At higher normal forces (*F_z_* = 3 N), the wear is considerably greater than at *F _z_*= 1 N, regardless of the surface type or the lubrication conditions. This is evident in the wear surface area and the ball’s wear radius. For the force of *F_z_* = 3 N, ball wear in dry conditions is significantly higher than in oil-lubricated conditions (see Table 10). However, for the force of *F_z_* = 1 N, the wear ratio between the dry and lubricated conditions decreases markedly, and, in some cases, it is even reversed. This ratio also declines as the surface roughness increases (surfaces D and E). When comparing ball wear levels in dry and lubricated contact, a reduction in the ratio of the dry wear to the oil-lubricated wear can be observed with an increasing surface roughness and a decreasing load. The observations indicate that the wear levels are comparable for surfaces D and E, regardless of the contact conditions (dry or lubricated). In analyzing the influence of the surface texture orientation, no clear difference in wear levels was observed between the X and Y directions. The ball–base pair (surface A) exhibits the highest wear compared to the other surfaces regardless of the testing conditions.

Theoretical analysis of the contact stresses showed that the contact circle diameter for 1 N and 3 N is 51 µm and 73 µm, respectively. The contact level resulting from the trace diameter on the counter-specimen surface indicates a much higher value than the theoretical value, regardless of the load and lubrication conditions. However, it can be seen again that the contact level for samples D and E is similar regardless of the lubrication conditions.

In conclusion, the analysis shows that the surface texture’s periodicity and characteristics significantly impact the steel ball’s wear. Textured surfaces exhibit lower wear than raw surfaces, particularly under oil-lubricated conditions. Lubrication substantially reduces the wear in all cases, though the effect of the texture orientation (X and Y) is marginal. The results suggest that an appropriately designed surface texture can improve the tribological properties of materials by reducing the wear, especially in the presence of lubrication.

### 3.8. Frictional Performance of Laser-Textured Steel

**Figure 10:**
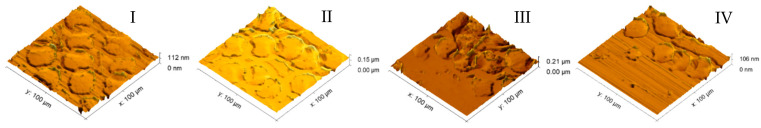
Stages of changes on the surface of sample E during the wear test—conditions: *F_z_* = 3 N and dry contact: I—before the wear test, II—the stage of damage to the roughness peaks, III—the stage of closing the depressions and IV—the stage of destruction of the microstructure of the surface layer.

The observations and analyses indicate that the nature of the contact and frictional processes is influenced by the following two types of surface layer structures: micro- and nanostructures. Moreover, depending on the complexity of the structure, lubrication conditions, and the stage of the friction process, different characteristics of changes are observed. Nanoscale contact occurs exclusively during the “initial” contact phase, before the stabilization of friction, for surfaces A, B, and C. The lower the contact stresses, the longer the nanoscale contact persists, and the presence of lubrication significantly prolongs this process. The research observations analyzed in the previous sections suggest friction’s complex, texture-dependent nature. In the example of the textured surface D in dry conditions and at a load of *F_z_* = 3 N, the changes in the ball–surface contact and the level of wear are noticeable. As a result, the roughness peaks are cut off (the transition from stage I to II; see Figure 10). The ball–surface contact increases and the wear products fill the roughness valleys. The presence of debris supports the friction process by creating a lubricant film. The intensification of the process (the transition from stage II to III) causes the creation of a new surface topography. As a result of this change, the nature of the wear also changes, transforming into abrasive wear (surface grooves) with elements of adhesive wear. In the presence of oil, the wear is limited due to the mixed and hydrodynamic lubrication phenomenon. In the case of surfaces A, B, and C, the observations showed that the wear mechanisms under specific parameters do not differ significantly from each other. Under dry conditions, adhesive wear with elements of abrasive wear dominates (Figure 10). It is worth noting that, for surfaces B and C, the ball primarily experienced abrasive wear, which may be due to changes in the hardness of the surface layer (Figure 6). Due to the multi-point contact of the ball with the surface, debris generated from the contact is deposited across the entire contact width. In some cases, fragments of material act as a third body in the ball–surface contact, reducing the system to a three-body model. This results in numerous scratches in the material excess and the raw surface (Figure 6).

**Figure 11:**
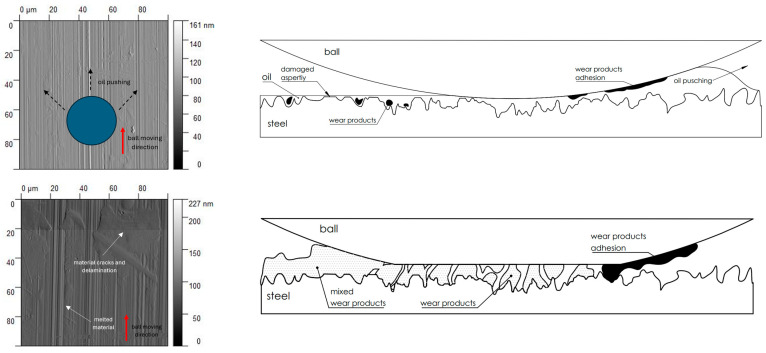
Frictional performance of raw, random and quasi-random in dry and lubricated contact.

In the presence of silicone oil, the level of wear decreases, altering the nature of the changes. The abrasive wear mechanism dominates, resulting in plastic deformations in the scratches oriented parallel to the ball’s movement direction. The small amount of debris also confirms this. Silicone oil, by creating a film, minimizes the friction process. However, during the ball’s movement, the oil is pushed out of the contact area, reducing its impact (see Figure 11). The size of the surface roughness valleys does not provide sufficient space for the oil to settle continuously and create suitable hydrodynamic conditions. Furthermore, wear products remaining in the contact zone exacerbate the wear process.

**Figure 12:**
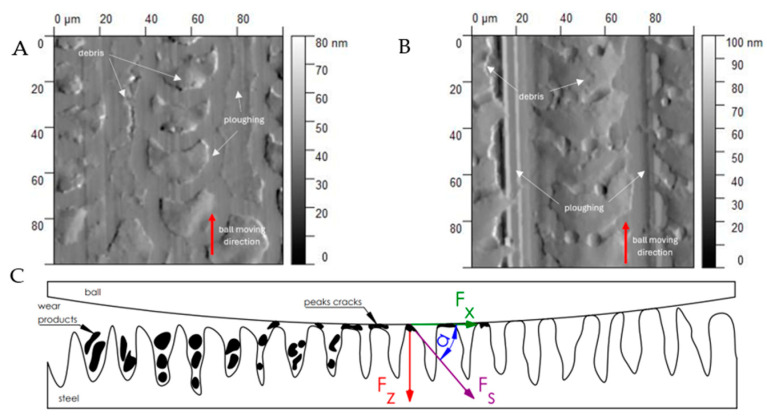
Distribution of oil cavities on the laser-structured surfaces: quasi-periodic and periodic (**A**,**B**) and stress distribution in the ball–surface oil contact zone (**C**).

In the case of surfaces D and E, during the absence of lubrication, the direct analysis of the wear cross-section indicates the dominance of abrasive wear, with plastic deformations oriented in the direction of the ball’s movement (Figure 12). Moreover, this phenomenon is independent of the direction of the movement relative to the surface texture, as confirmed by the analysis of the scratch width (Table 5). It is worth noting that the degradation of the original surface topography occurs gradually (Figure 10), which makes the wear process more complex and challenging to characterize unequivocally. As the surface degradation progresses, there is a potential increase in the ball–surface contact area. This may indicate that the progression of surface damage is not linear but depends on the area of the original contact and the subsequent surfaces derived from the topography cross-section. The resultant shear force *Fs* causes peak cracks, and the intensity of these cracks increases with the rise in the COF, corresponding to an increase in the penetration angle of *F_s_* − α. Fragments of the ball accumulate in the spaces between the roughness peaks until a new surface structure is formed, which is not characterized by periodicity.

**Figure 13:**
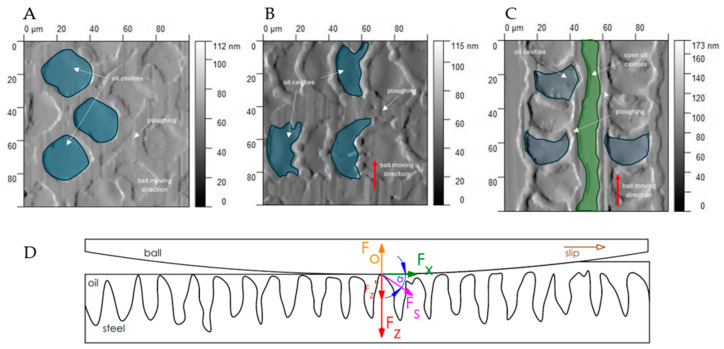
Distribution of oil cavities on the laser-structured surfaces: quasi-periodic and periodic (**A**–**C**) and stress distribution in the ball–surface oil contact zone (**D**).

In the presence of silicone oil, the frictional performance of the laser-textured surfaces D and E is characterized by the most excellent stability and the least wear (Figure 13). In this case, the oil accumulated in the spaces between the roughness peaks, as well as in the form of a thin lubricating layer, allows for the transfer of loads through the action of hydrodynamic forces.

The fluid pressed into the spaces between the peaks increases the hydrodynamic lubrication, thus reducing the COF. The study by Kholsa et al. supports this [52], which describes the same phenomena in porous substances. The load *F_z_* is reduced by the fluid response *Fo*, resulting in a shear force composed of Fz’ and Fx. Moreover, the force Fx is not a direct tangential force resulting from ball–surface contact but from the ball slipping on the oil film. Liu et al. made similar observations [53]. In that case, the distance between the micro-pits was 500 µm, whereas, in this study, it was about 100 µm. Most importantly, it was observed that the best solution is the application of structures where oil cavities with a closed character can form (Figure 13). This prevents the oil from escaping under high pressure, thus ensuring the formation of a stable oil film.

## 4. Conclusions

Laser surface texturing of AISI 321 stainless steel enables the effective control of its tribological properties through several of the following mechanisms:

- The phase transformation of austenite into martensite, occurring during the process, increases the surface hardness, significantly improving the wear resistance.
- The texturing enhances the surface’s hydrophobicity, minimizing the direct contact between bodies and thereby reducing the material wear.
- The creation of regular structures and their periodicity stabilizes the contact and alters the distribution of contact stresses, which is crucial for improving the durability of components operating under demanding conditions.
- The periodic structures promote the formation of oil reservoirs, enabling operation in mixed lubrication (ML) or hydrodynamic lubrication (HL) regimes, further enhancing the lubrication efficiency and extending the service life of components.
- The change in the friction from sliding to rolling due to the influence of the surface texture on the hydrodynamic effects, as evidenced by the changes in the friction coefficient value.
- Laser-induced periodic surface structures (LIPSSs) further optimize the tribological properties, making this technology a promising tool for surface modification in various industrial applications.

The research hypothesis assumed that structuring AISI 321 steel, aimed at periodizing the surface shape, enables the improvement of its tribological properties, which the results have confirmed. However, it is also evident that friction processes will significantly depend on the contact conditions, both with and without lubrication. Moreover, it depends on the wear stage due to the variability of the contact geometry over time. It was observed that each ball passed during wear tests resulted in partial changes to the surface topography. Nanoscale structures were destroyed after the first pass, while microscale structures could persist for longer, depending on the stress, direction, and lubrication. The results confirm that laser structuring enables the modification of the tribological properties of AISI 321 steel by modifying many features of the surface layer.

## Figures and Tables

**Table 1:** Basic parameters implemented during the tests: *F_z_*—pressure force, *p_max_*—maximum stresses in the contact zone, *t_max_*—maximum shear stresses in the contact zone, *z*—depth of maximum stresses, *d*—theoretical contact zone diameter, *v*—movement velocity, *s*—movement range, and *t*—test time.

Parameter	*F_z_* [N]	*p_max_* [MPa]	*τ_max_* [MPa]	*z* [µm]	*d* [µm]	*v* [mm/s]	*s* [mm]	*t* [s]
Value	1	744	230	12	51	10	5	360
	3	1073	332	18	73	10	5	360

**Table 2:** Surface topography parameters for the samples before the tribology test.

Name	Unit	Sample				
		A	B	C	D	E
Sa	µm	0.24	0.25	0.28	0.38	1.00
Sq	µm	0.31	0.34	0.34	0.49	1.23
Sp	µm	6.04	3.23	1.32	2.96	9.38
Sv	µm	42.4	33.8	1.6	7.7	3.1
Sz	µm	48.5	37.0	3.0	10.6	12.5
S10z	µm	4.44	9.52	2.48	5.60	9.29
Ssk		−2.01	−5.25	−0.06	−0.96	0.44
Sku		412	494	3	14	3
Sdq		0.04	0.10	0.04	0.10	0.22
Sdr	%	0.10	0.34	0.08	0.45	2.42

**Table 3:** Comparison of the basic roughness parameters for the surfaces of the samples and the scratch surfaces after the tribological testing.

Surface			Random			Quasi-Random			Quasi-Periodic			Periodic		
Parameter	*F_z_*	Lubricant	*Y*	*X*	Raw	*Y*	*X*	Raw	*Y*	*X*	Raw	*Y*	*X*	Raw
Sa	3	dry	0.30	0.23	0.17	0.32	0.23	0.18	0.47	0.32	0.46	0.82	0.76	1.00
		oil	0.13	0.28		0.14	0.22		0.49	0.47		0.98	0.95	
	1	dry	0.24	0.22		0.34	0.21		0.33	0.48		1.04	1.00	
		oil	0.15	0.27		0.14	0.18		0.38	0.46		0.62	0.98	
Sq	3	dry	0.45	0.30	0.21	0.47	0.28	0.23	0.59	0.44	0.65	1.00	0.93	1.22
		oil	0.17	0.35		0.18	0.29		0.61	0.58		1.14	1.10	
	1	dry	0.33	0.27		0.43	0.35		0.43	0.60		1.23	1.23	
		oil	0.20	0.34		0.18	0.25		0.48	0.59		0.82	1.18	
Sz	3	dry	9.67	1.80	2.00	3.00	1.17	3.90	5.38	6.60	13.60	10.90	4.90	7.67
		oil	1.22	1.39		1.60	1.68		3.10	4.22		6.38	3.34	
	1	dry	3.36	1.05		3.40	2.2		1.55	5.62		3.79	7.70	
		oil	1.70	1.29		0.65	4.94		3.36	3.90		12.00	3.34	
Ssk	3	dry	3.23	0.52	0.52	0.76	0.13	0.36	−0.18	1.18	1.76	0.47	2.55	0.31
		oil	0.44	0.23		−0.20	0.98		−0.58	−0.33		−0.04	0.08	
	1	dry	0.93	0.09		0.33	1.2		−0.54	−0.15		0.75	0.24	
		oil	0.94	0.26		−0.51	1.52		−0.47	−0.02		1.37	0.20	
Sku	3	dry	48.10	3.70	4.00	8.70	3.00	7.00	3.38	13.00	24.00	6.35	−0.07	2.68
		oil	4.87	2.92		3.80	4.84		3.00	2.94		2.33	2.14	
	1	dry	9.27	2.95		4.20	4.6		3.29	4.39		2.10	2.25	
		oil	6.69	2.91		4.22	24.00		3.30	3.43		17.80	2.17	
Vvc	3	dry	0.46	0.25	0.25	0.48	0.35	0.27	0.67	0.51	0.67	1.16	1.16	1.65
		oil	0.20	0.34		0.21	0.36		0.62	0.63		1.40	1.45	
	1	dry	0.36	0.26		0.48	0.29		0.48	0.68		1.60	1.70	
		oil	0.23	0.45		0.20	0.26		0.50	0.66		0.93	1.66	

**Table 4:** Effect of the lubrication, load, and direction on the average COF values for the analyzed surfaces.

*F_z_* [N]		1		3	
Lubricant		Dry	Oil	Dry	Oil
Surface	B				
Direction	Y	0.34 ± 0.04	0.9 ± 0.23	0.12 ± 0.01	0.27 ± 0.05
	X	0.85 ± 0.08	0.79 ± 0.08	0.15 ± 0.01	0.13 ± 0.01
Surface	C				
Direction	Y	0.37 ± 0.04	2.29 ± 0.4	0.17 ± 0.04	0.33 ± 0.08
	X	0.93 ± 0.15	0.85 ± 0.08	0.12 ± 0.01	0.12 ± 0.01
Surface	D				
Direction	Y	0.41 ± 0.03	2.15 ± 0.25	0.15 ± 0.01	0.26 ± 0.06
	X	1.14 ± 0.15	0.15 ± 0.01	0.16 ± 0.01	0.16 ± 0.01
Surface	E				
Direction	Y	1.33 ± 0.52	1.22 ± 0.1	0.16 ± 0.008	0.24 ± 0.06
	X	0.92 ± 0.14	0.17 ± 0.01	0.19 ± 0.02	0.17 ± 0.02
Surface	A				
Direction	Y	0.28 ± 0.1	0.58 ± 0.05	0.76 ± 0.08	0.46 ± 0.11
	X				

**Table 5:** The width of the scratch formed during the friction tests.

*F_z_* [N]		3		1	
Surface	Direction	Lubricant			
		Dry	Oil	Dry	Oil
		Width, µm			
B	X	325	117	188	72
	Y	366	141	155	110
C	X	299	139	135	93
	Y	305	156	180	93
D	X	278	228	222	160
	Y	271	216	266	161
E	X	312	297	240	227
	Y	367	308	204	169
A		431	357	387	120

**Table 6:** The surface roughness profile of sample B with the contact range indicated (red rectangle), and the characteristic features of the scratch geometry, the A—groove (red arrow) and B—material transfer (black arrow).

Scratch View	Surface Roughness Profile	Conditions
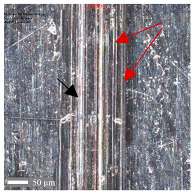	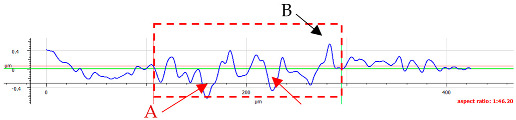	*F_z_* = 1 N dry
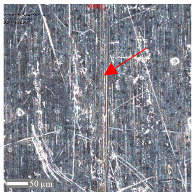	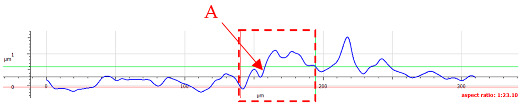	*F_z_* = 1 N oil
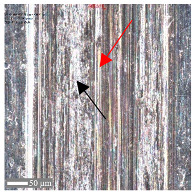	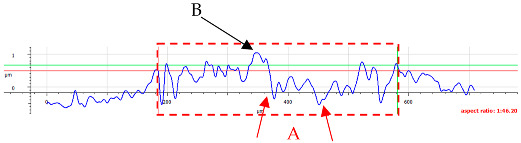	*F_z_* = 3 N dry
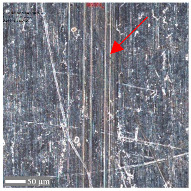	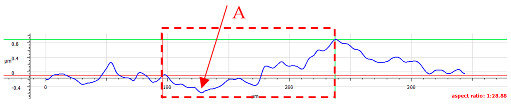	*F_z_* = 3 N oil

**Table 7:** The surface roughness profile of sample C with the contact range indicated (red rectangle), and the characteristic features of the scratch geometry, the A—groove (red arrow) and B—material transfer (black arrow).

Scratch View	Surface Roughness Profile	Conditions
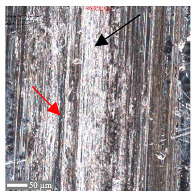	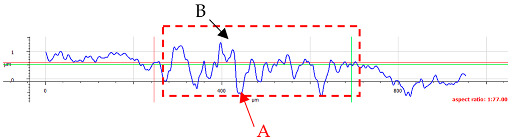	*F_z_* = 1 N dry
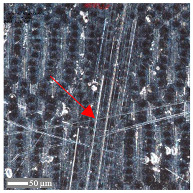	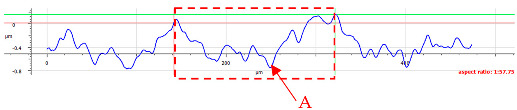	*F_z_* = 1 N oil
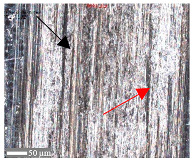	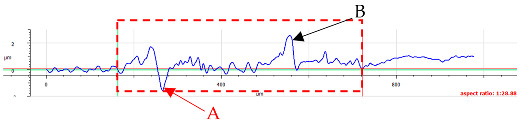	*F_z_* = 3 N dry
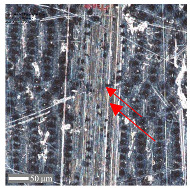	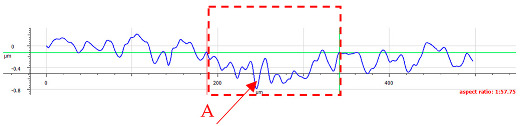	*F_z_* = 3 N oil

**Table 8:** The surface roughness profile of sample D with the contact range indicated (red rectangle) and the damage to the roughness peaks (red arrow).

Scratch View	Surface Roughness Profile	Conditions
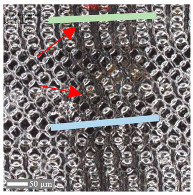	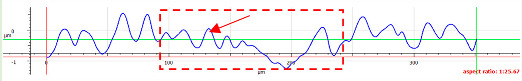	*F_z_* = 1 N dry
	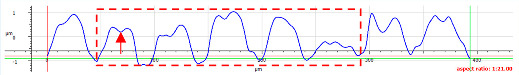	
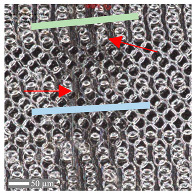	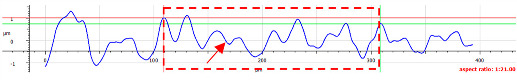	*F_z_* = 1 N oil
	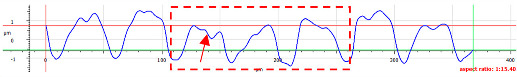	
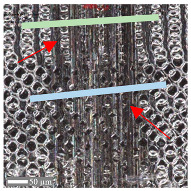	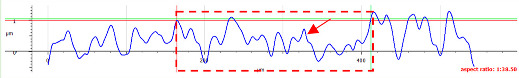	*F_z_* = 3 N dry
	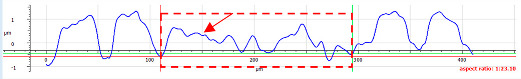	
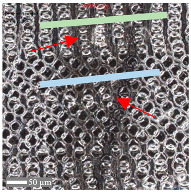	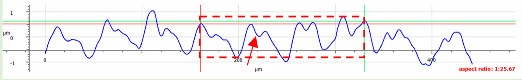	*F_z_* = 3 N oil
	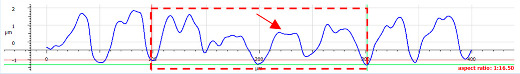	

**Table 9:** Surface roughness profile of sample E with the contact range indicated (red rectangle) and the damage to the roughness peaks (red arrow).

Scratch View	Surface Roughness Profile	Conditions
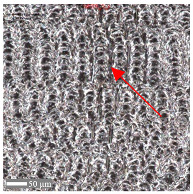	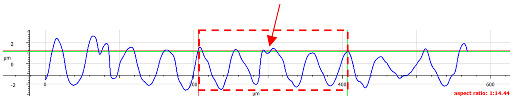	*F_z_* = 1 N dry
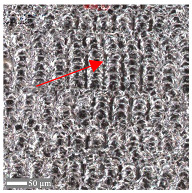	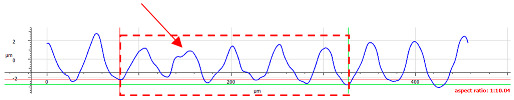	*F_z_* = 1 N oil
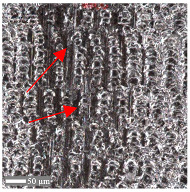	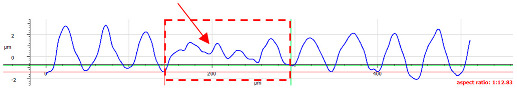	*F_z_* = 3 N dry
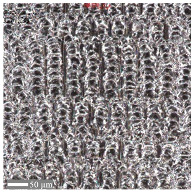	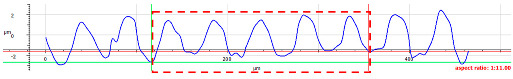	*F_z_* = 3 N oil

**Table 10:** Carbon steel ball wear level.

*F_z_* [N]		3		1		3		1	
Surface	Direction	Lubricant							
		Dry	Oil	Dry	Oil	Dry	Oil	Dry	Oil
		*A*, µm × 10^5^				*d*, µm			
B	X	1.59	0.21	0.48	0.11	450	162	247	121
	Y	1.21	0.20	0.25	0.18	392	160	180	150
C	X	3.19	0.47	0.88	0.18	637	245	334	151
	Y	2.98	0.18	0.17	0.23	616	150	146	483
D	X	0.75	0.44	0.33	0.31	309	236	205	199
	Y	0.39	0.46	0.30	0.52	223	241	194	257
E	X	0.96	0.83	0.60	0.61	350	326	276	279
	Y	0.76	0.81	0.55	0.84	311	321	264	328
A		3.67	1.11	1.38	1.42	342	683	377	419

## Data Availability

The original contributions presented in the study are included in the article, further inquiries can be directed to the corresponding author.
